# What Matters 2 Kids: a mixed-methods protocol for developing a culturally-responsive illustrated well-being measure for Aboriginal and Torres Strait Islander children

**DOI:** 10.1136/bmjopen-2025-115255

**Published:** 2026-06-18

**Authors:** Kate Mallory Anderson, Kirsten Howard, Michelle Dickson, Alana Gall, Darren Garvey, Martin Howell, Maryanne Theobald, Brendan James Mulhern, Rosalie Viney, Gail Garvey

**Affiliations:** 1 Yardhura Walani Centre, Australian National University, Acton, Australian Capital Territory, Australia; 2 First Nations Cancer and Wellbeing Research Program, School of Public Health, The University of Queensland, Brisbane, Queensland, Australia; 3 Leeder Centre for Health Policy, Economics and Data, Faculty of Medicine and Health, University of Sydney, Sydney, New South Wales, Australia; 4 The Poche Centre for Indigenous Health, Faculty of Medicine and Health, University of Sydney, Sydney, New South Wales, Australia; 5 National Centre for Naturopathic Medicine, Faculty of Health, Southern Cross University, East Lismore, New South Wales, Australia; 6 Curtin School of Population Health, Curtin University, Perth, Western Australia, Australia; 7 School of Education, Faculty of Creative Industries, Education and Social Justice, Queensland University of Technology, Brisbane, Queensland, Australia; 8 The Centre for Health Economics Research and Evaluation (CHERE), University of Technology Sydney, Ultimo, New South Wales, Australia

**Keywords:** Adolescent, Community child health, MENTAL HEALTH

## Abstract

**Abstract:**

**Introduction:**

Aboriginal and Torres Strait Islander children are the future custodians of country, culture and community for the world’s oldest living cultures. However, they continue to experience significant disadvantage as a legacy of British colonisation. Failure to address the systemic inequities experienced by Aboriginal and Torres Strait Islander children infringes on their rights to health, education, safety and cultural identity. Despite this, there has been limited focus on recognising and measuring the cultural and personal strengths that support their well-being. This study aims to co-design a culturally grounded, illustrated self-report well-being measure for Aboriginal and Torres Strait Islander children aged 5–11 years, focusing on the aspects of life that nurture their strength and well-being.

**Methods and analysis:**

The project will employ a five-phase mixed methods design, guided by principles of co-design and best practices to develop culturally and developmentally responsive measures with Aboriginal and Torres Strait Islander children. Extending existing methods, we will pioneer a new approach to develop measures enhanced with culturally-responsive pictures co-designed with Aboriginal and Torres Strait Islander communities.

**Ethics and dissemination:**

Ethics approvals have been obtained from the University of Queensland Human Research Ethics Committee (2023/HE000607), Western Australian Aboriginal Health Ethics Committee (WAHREC1260), Far North Queensland Human Research Ethics Committee (2023/QCH/99346), Aboriginal Health and Medical Research Council of New South Wales (2140/23), Aboriginal Health Council of South Australia (04–23-1063), Menzies School of Health Research and Northern Territory Department of Health Human Research Ethics Committee (HREC 2023–4608) and the Australian National University Human Research Ethics Committee (H/2024/0914), as well as relevant Departments of Education. The final WM2K (*What Matters 2 Kids*) results will be published in peer-reviewed journals, presented at conferences and disseminated to partner organisations and the broader sector via strategic networks with Aboriginal and Torres Strait Islander organisations, government bodies and non-government agencies. Children will receive study communications via partner organisations in age-appropriate formats.

**STRENGTHS AND LIMITATIONS OF THIS STUDY**

- The five-phase mixed-methods design integrates co-design principles, qualitative concept elicitation, culturally-responsive artwork co-design, psychometric validation and preference-based scoring, providing a robust methodological framework for developing a culturally grounded well-being measure with Aboriginal and Torres Strait Islander children.
- Aboriginal and Torres Strait Islander leadership is embedded throughout all research phases via a majority-Indigenous Co-Design Working Group, strengthening governance and cultural integrity in the development process.
- Recruiting sufficiently large, diverse samples of Aboriginal and Torres Strait Islander children across metropolitan, rural and remote areas within proposed timelines is a potential methodological limitation, and contingency plans are in place to manage this.
- The feasibility of a 30-minute online survey for children aged 5–11 years has not been empirically established in this context, and adaptations to reduce cognitive load will be piloted and reported.

##  Background

Aboriginal and Torres Strait Islander children are the future custodians of the world’s oldest living cultures, with ancestral, cultural and spiritual connections to the lands and waterways now known as Australia that span over 65 000 years.^1^ These enduring ties provide access to rich knowledge systems and relational networks that support well-being.^2^

Despite Australia’s endorsement of the United Nations Convention on the Rights of the Child^3^ and the United Nations Declaration on the Rights of Indigenous Peoples,^4^ Aboriginal and Torres Strait Islander children continue to experience systemic inequities that infringe on their rights to health, education, safety and cultural identity.^5^ These inequities largely stem from the ongoing impacts of colonisation, which continue to cause significant challenges such as intergenerational trauma, systemic racism and disruption to culture and identity. Compared with non-Indigenous Australian children who benefit from some of the highest well-being standards globally,^6^ Aboriginal and Torres Strait Islander children experience disproportionate disadvantage,^7^ manifesting in higher rates of bullying, unfair treatment and suicide, which is the leading cause of death among children aged 5–17 years.^5^ Addressing these disparities is essential for breaking cycles of disadvantage, fostering long-term benefits for individuals, communities and society, and for upholding Aboriginal and Torres Strait Islander children’s rights.^3 4^

### The need for culturally grounded well-being measurement

Accurate data collected consistently and at scale is needed to inform and evaluate programmes and policies to more effectively support Indigenous children’s well-being and uphold their rights.^5 8^ However, current child well-being indicators are inadequate for this purpose. Existing measures often fail to capture aspects of life that are meaningful to Aboriginal and Torres Strait Islander children, limiting their relevance for policy and funding decisions.^8^

Well-being is commonly defined as a state of equilibrium achieved when an individual’s psychological, social and physical resources are sufficient to meet the challenges they face.^9^ For Aboriginal and Torres Strait Islander peoples, however, well-being is deeply relational; grounded in connections to community, culture and country.^2 10 11^ Dominant biomedical models often overlook these dimensions and fail to reflect Indigenous worldviews and strengths. While acknowledging the linguistic and cultural diversity among Aboriginal and Torres Strait Islander communities, conceptions of well-being are commonly holistic, multidimensional and closely tied to relationships with family, community, culture and country.^210^,^12^

Although a few mental health tools have been developed for Aboriginal and Torres Strait Islander children, none offer a comprehensive, culturally grounded and nationally applicable measure of well-being.^13^ Moreover, these tools do not readily support economic evaluation or policy-relevant decision making. How children understand well-being differs significantly from the understandings of adults and adolescents,^14 15^ but research in this area remains limited, particularly for Aboriginal and Torres Strait Islander children. To develop a meaningful and robust measure, it is essential to centre the voices of Aboriginal and Torres Strait Islander children, recognising them as experts in their own lives. Measures developed without their input will likely misrepresent their needs and priorities.^16^

### The role of visual support in child self-report

Research has consistently shown that younger children experience difficulty completing surveys using conventional text-only formats.^17 18^ Incorporating visual elements alongside written content can enhance children’s comprehension and self-completion of surveys and measures.^19 20^ Visual representations support word recall, improve focus and aid conceptual understanding while requiring less cognitive processing than text alone.^19 20^ Additional benefits of combining imagery with text include capturing and maintaining young children’s engagement, which may lead to higher quality responses and improved survey completion.^21^ Despite these recognised benefits, to our knowledge, no well-being measures have incorporated culturally-responsive artwork co-designed with Aboriginal and Torres Strait Islander children to support both comprehension and cultural connection.

### The what matters 2 kids study

To address these gaps in well-being measurement, our team has been working for the past 10 years on the *What Matters programme*, which is a multidisciplinary research collaboration that is developing a suite of well-being measures specifically for Aboriginal and Torres Strait Islander peoples over the life course.^1022^,^24^ As part of this programme, the *What Matters 2 Kids* (WM2K) project will co-design a nationally relevant, strengths-based, preference-scored illustrated well-being measure for Aboriginal and Torres Strait Islander children aged 5–11 years. This age span was chosen to extend our other *What Matters* measures (*What Matters 2 Adults* (WM2A) and *What Matters 2 Youth* (WM2Y) for adults and youth), which together measure well-being for Aboriginal and Torres Strait Islander peoples 12 years and older.^23 24^ Grounded in children’s experiences, values and cultural contexts, this measure will support evidence-based decision making in clinical, educational and policy settings.

Our multidisciplinary team will work in partnership with communities and organisations to ensure children’s voices are central to the process. Through collaborations with trusted service providers, we will facilitate safe, respectful and meaningful engagement. Uniquely, the WM2K measure will feature culturally-responsive artwork co-designed with Aboriginal and Torres Strait Islander children and artists from diverse communities across Australia. This approach ensures the resulting measure is both culturally resonant and practically useful, advancing both the science of well-being measurement and the rights of Aboriginal and Torres Strait Islander children to define and assess their own well-being.

## Methods

The project will use a five-phase mixed methods design, following principles for co-design with Aboriginal and Torres Strait Islander people,^25^ established mixed-methods approaches for preference-based measure development^26^ and novel methods to develop culturally-responsive pictures to accompany measure items, co-designed with Aboriginal and Torres Strait Islander communities. We will draw on processes successfully applied by our team in previous projects (National Health and Medical Research Council (NHMRC) GNT 1125434; MRFF1199854) and other studies.^27^,^29^ Our *WM2A and WM2Y* projects have been successfully completed,^23 24^ demonstrating the feasibility and acceptability of our research methods. Extending existing methods, we will pioneer new methods to develop measures enhanced with culturally-responsive pictures co-designed with Aboriginal and Torres Strait Islander communities. These methods will ensure the artwork is developed through co-design with children from diverse communities and refined through community feedback, validate the pictorially-supported measure with artwork as an integral component and centre Aboriginal and Torres Strait Islander children’s voices in both content and visual representation in the measure. The principles and practices for conducting co-design projects with Aboriginal and Torres Strait Islander people^25^ will be applied throughout the project. We will conduct this study in accordance with the NHMRC Guidelines for the Ethical Conduct in Aboriginal and Torres Strait Islander Health Research^30^ and the AIATSIS (Australian Institute of Aboriginal and Torres Strait Islander Studies) Code of Ethics for Aboriginal and Torres Strait Islander Research.^31^

The study commenced in January 2024 and is expected to be completed by December 2027. Data collection for Phase 2 (Collaborative Exploration) is currently underway, with Art and Yarning sessions being conducted across partner sites nationally.

The five phases of the project are:

- Phase 1: establish a co-design working group;
- Phase 2: collaborative exploration to identify foundations of well-being;
- Phase 3: co-design of culturally responsive pictorial elements;
- Phase 4: develop and validate the pictorially-supported descriptive system; and
- Phase 5: develop a preference-based scoring system.

### Research team

The research team includes Aboriginal and Torres Strait Islander Peoples (AG, DG, MD, GG), qualitative researchers (KMA, AG, DG, MD, MT, GG) and health economists (KH, MH, BJM, RV) working in Aboriginal and Torres Strait Islander health research. As a research team, we are conscious of the importance of our backgrounds and perspectives, and reflexively consider how such factors may influence our approach to research.^32 33^

### Indigenist methodology

The current study is described in detail below as per the requirements of the *Consolidated criteria for strengthening reporting of health research involving Indigenous peoples* (the CONSIDER statement).^34^ The study will employ a strength-based, Indigenist research approach and methods to ensure that Aboriginal and Torres Strait Islander children’s voices guide all aspects of the research.^35 36^ Given the enduring process of colonial disempowerment and marginalisation of Australia’s Aboriginal and Torres Strait Islander peoples, the use of Indigenist methodologies and a strength-based approach strive to avoid further subjugation of these groups. Our research team has extensive experience in using Indigenist research methodologies and methods^35 36^ to ensure that our work contributes to improving the outcomes and experiences of Aboriginal and Torres Strait Islander peoples. Our approach will be guided by the following principles: ensuring Aboriginal and Torres Strait Islander voices and perspectives are prioritised and privileged throughout all aspects of the research process and our work; building Aboriginal and Torres Strait Islander peoples’ research capacity and developing future research leaders; and facilitating collaboration through engaging and connecting with a range of key stakeholders.

Co-design Working Group (CWG) members are identified through consultation with partner organisations, prioritising individuals with relevant community standing, cultural authority and expertise. Roles and responsibilities are agreed at project commencement, with members participating across all phases and onboarding provided at project initiation. The CWG operates under agreed governance principles that include equitable power distribution, transparent decision making and respect for community priorities. Where consensus cannot be reached, decisions are escalated through respectful dialogue facilitated by the Aboriginal and Torres Strait Islander lead investigators, with community priorities taking precedence over prespecified research plans. Conflict resolution processes are documented in the project’s governance framework. Indigenist principles are operationalised in practice through: privileging Aboriginal and Torres Strait Islander voices in all analytical interpretations via Collaborative Yarning; ensuring all recruitment, consent and data collection is led or co-led by Aboriginal and Torres Strait Islander researchers; and centring community review at critical decision points in item development, artwork refinement and psychometric interpretation. Aboriginal and Torres Strait Islander leadership and governance throughout the project is a foundational element of Indigenist methodologies and equitable processes. This includes a CWG established on project commencement, consisting of a majority of Aboriginal and Torres Strait Islander knowledge holders and representatives from youth organisations, service providers, schools and community-controlled health services.

### Community involvement

Patient and Public Involvement: Aboriginal and Torres Strait Islander children, families, elders and community organisations were involved in the design and planning of this study through the CWG, established prior to project commencement. The CWG-guided decisions about research questions, methods, recruitment approaches and cultural protocols. Aboriginal and Torres Strait Islander Community Researchers are co-facilitators in all research activities, reflecting the study’s foundational commitment to Indigenous Data Sovereignty and self-determination.

Aboriginal and Torres Strait Islander peoples’ leadership and governance is foundational to this research. The CWG comprising majority Aboriginal and Torres Strait Islander knowledge holders and representatives from youth organisations, service providers, schools and community-controlled health services was established prior to project commencement and guides all aspects of the research process. Aboriginal and Torres Strait Islander children are central to every phase as active co-researchers and knowledge holders: approximately 150 children will participate in Art and Yarning sessions to identify well-being foundations (Phase 2), 10–20 will refine items through think-aloud yarns (Phase 2b), 15–20 per community will co-design culturally-responsive artwork with Aboriginal and Torres Strait Islander artists (Phase 3) and approximately 300 and 400 children will participate in validation and preference scoring activities respectively (Phases 4–5). Aboriginal and Torres Strait Islander Community Researchers will co-facilitate all research activities and contribute to collaborative analysis processes, while cultural knowledge holders will provide guidance on cultural protocols. Research priorities and methods were informed by our team’s decade-long partnership with Aboriginal and Torres Strait Islander communities through the *What Matters* programme and ongoing CWG consultation. Children will be reimbursed with art materials, all sessions are designed to be culturally safe and strengths-based with experienced Aboriginal and Torres Strait Islander Research Officers co-facilitating, and activities are conducted in partnership with trusted community organisations. Study findings will be communicated to children through age-appropriate formats via partner organisations, and the final measure will be freely accessible to support improved well-being assessment for Aboriginal and Torres Strait Islander children.

### Phase 1: Establish co-design working group

In Phase 1, prior to project commencement, we will establish the CWG to embed the principles and best practices of co-design throughout the project. Ensuring appropriate governance structures are in place to guide and monitor the project is essential to co-design with Aboriginal and Torres Strait Islander peoples^37^ and adequate time and resources will be allocated to facilitate successful and respectful co-design.^37^ Regular and sustained communication channels endorsed by the community are also essential for co-design with Aboriginal and Torres Strait Islander peoples.^37^ We will work with the CWG to establish culturally safe and respectful communication using modes, platforms and formats that align with Aboriginal and Torres Strait Islander preferences and protocols.^37^ Iterative communication loops between stakeholders will enable key concepts and features to develop in culturally appropriate ways.^37^

### Phase 2: Collaborative exploration to identify foundations of well-being

In Phase 2, guided by the CWG, we will use culturally responsive art-based qualitative methods to identify the foundations of well-being for Aboriginal and Torres Strait Islander children. We will then use co-design practices to develop strengths-based well-being items that reflect these identified components of well-being.

#### Phase 2a: Collaborative exploration of children’s well-being using art and yarning

We will conduct a large, national qualitative study to gather the views of Aboriginal and Torres Strait Islander children about what is important to, and impacts on, their well-being. An adapted form of the Draw, Write, Tell method,^38^ termed the *Art and Yarning* method, will be used to investigate and understand the parts of life that contribute to Aboriginal and Torres Strait Islander child well-being. Draw, Write, Tell is a creative research technique often used in research involving children. It combines three activities: children are asked to draw pictures related to the research topic; they write about their drawings or the topic; and they verbally explain their drawings and writings.^38^ This method allows children to express their thoughts and feelings in multiple ways, accommodating different communication preferences and abilities. It has been used in various fields, including health, social care and education, to gather rich, qualitative data from children.^38 39^ *Yarning* is an Aboriginal and Torres Strait Islander culturally grounded method of communication, and involves sharing knowledge, listening, interpreting, re-interpreting and collective sense-making. Yarning in a research context privileges Aboriginal and Torres Strait Islander knowledges by grounding data and data collection in Aboriginal and Torres Strait Islander values and traditions.^40^ Yarning in the research context comprises informal social discussion and more formal research-focused interaction. A culturally significant feature of yarning is its emphasis on relatedness within the research context between researcher and the knowledge holders.^40^ Yarning develops familiarity and trust between parties who may not be known to each other while permitting pre-existing relationships and the knowledge held within these to inform and influence the conduct of the yarn.^40^

##### Knowledge holders, recruitment and data collection

Approximately 15 Art and Yarning group sessions will be conducted, each with around 10 children aged 5–11 years (n=150), recruited via partner organisations across Australia. Separate Art and Yarning sessions with younger/older children will be run where possible. Art and Yarning and yarns will be co-facilitated by one of our experienced Aboriginal and Torres Strait Islander Research Officers. A purposive sampling approach will be used to ensure maximum diversity (eg, age, sex and remoteness) in the sample. Children will be acknowledged for their time via gifting the art supplies that they use in the study. The sample size and approach are consistent with our previous studies. Inclusion criteria: Aboriginal and Torres Strait Islander children aged 5–11 years who are able to participate in group art-based activities. Children with complex communication needs will be accommodated where possible through adaptation of activities in consultation with carers and community partners. Children who are not Aboriginal and Torres Strait Islander, or who fall outside the age range, will be excluded. Children will be recruited through partner organisations including community-controlled health services, schools and youth organisations. Prior to participation, written informed consent will be obtained from parents or legal guardians. Children will provide verbal assent, with research officers confirming understanding and willingness before commencing each session. Children may withdraw at any time without consequence.^10 24^

##### Collaborative analysis

All sessions with children will be audio recorded, transcribed verbatim and uploaded into NVivo V.12^41^ for analysis. We will use an iterative Collaborative Yarning approach^42^ to analyse the transcripts and identify the components of well-being important to children, which will be used to co-design a set of draft strengths-based well-being items, an approach with which our team has extensive experience.^10 22^ This approach will be conducted through a series of group sessions involving Aboriginal and Torres Strait Islander researchers, Community Researchers and members of the CWG. The final set of items will be phrased appropriately for self-complete by children. Two versions of the measure may be developed if deemed appropriate in the think-aloud yarns in Phase 2b: one for older children (aged 8–11 years) and one for younger children (aged 5–7 years). Analysts include KMA, AG, DG, GG and MD. Cross-checking of coding will be conducted between at least two analysts to enhance rigour, with discrepancies resolved through discussion and consensus.

### Phase 2b: Refine well-being items and assess content and face validity

We will use a culturally appropriate think-aloud method^43^ developed by our team called think-aloud yarns^44^ to assess content validity of the items. These think-aloud yarns will elicit whether: (1) respondents report missing components of well-being, (2) items are difficult to understand and (3) there are topics or item phrasing that are appropriate for younger or older children (to guide the potential development of age-relevant versions if needed).

#### Knowledge holders, recruitment and data collection

Think-aloud yarns will be conducted with Aboriginal and Torres Strait Islander children (n=10–20), and will be audio recorded, transcribed verbatim and uploaded into NVivo V.12^41^ for analysis. The think-aloud yarns will take 30 min of children’s time to complete. The data will be analysed in two steps: (1) error coding of statements and (2) thematic analysis of statement descriptive data.^45 46^ This method has been used successfully in our adult study (NHMRC GNT1125434) and in the development of other measures.^47^ While standard think-aloud interviews have been used previously with children, to our knowledge, this method has not been used with Aboriginal and Torres Strait Islander children. Previous think-aloud studies with children have adapted the task to allow greater comprehension and alleviate cognitive load, especially for younger children.^48^,^51^ As our team has already adapted standard think-aloud interviews for Aboriginal and Torres Strait Islander adults—think-aloud yarns—we will build on this and pilot adaptations with a small number of children to assess which adaptation works best with Aboriginal and Torres Strait Islander children.^51^ Children will be recruited from those in Phase 2a who expressed interest in participating in future study phases and will be sampled to ensure maximum diversity (eg, age, sex and remoteness). Children will receive an art materials pack.

#### Collaborative analysis

The descriptive data will be triangulated against interviewer field journals, then thematically analysed to provide more in-depth information on the content and face validity of the statements. We will also use this method to inform the recommended age of suitability for self-complete by children. We will engage the CWG using Collaborative Yarning to seek guidance before finalisation of the items and the determination of whether age-relevant versions of the measure are warranted.

### Phase 3: Co-design of culturally responsive pictorial elements

In Phase 3, we will use co-design methods to create Aboriginal and Torres Strait Islander artwork to accompany each well-being item developed in Phase 2. The artwork will be taken back to communities for feedback and refinement, to ensure it reflects shared cultural themes while incorporating the diverse perspectives gathered through the co-design workshops. This process will ensure the measure is visually accessible, culturally grounded and meaningful to Aboriginal and Torres Strait Islander children from diverse communities and language groups.

#### Knowledge holder recruitment

We will recruit approximately 15–20 Aboriginal and Torres Strait Islander children from each of the diverse communities participating in the co-design workshops (informed by Phase 2b findings regarding appropriate age groups). An Aboriginal and Torres Strait Islander artist with experience across diverse communities will be engaged to lead the artwork development. The artist will be identified through consultation with partner organisations, prioritising those with experience working with children, creating culturally appropriate educational materials and who hold cultural authority to create artwork that resonates across diverse Aboriginal and Torres Strait Islander communities.

#### Co-design process

Using Art and Yarning methods established in Phase 2, we will facilitate collaborative co-design workshops in diverse communities where children work with the artist to explore visual representations for each well-being item. Children will be presented with the finalised well-being items from Phase 2, and yarning circles will be used to explore how children visualise and conceptualise each well-being component. Children will be engaged in creating initial sketches and artwork concepts that reflect their cultural knowledge, stories and artistic traditions. The artist will then synthesise ideas from across all community workshops to develop a unified artwork version that incorporates shared cultural themes and diverse perspectives. The artist will return to participating communities to present the draft artwork and gather feedback, refining the designs through iterative consultation with children, local elders (where appropriate) and the CWG to ensure the final artwork reflects cultural knowledge that resonates across diverse communities. The co-design workshops will take place over 1–2 weeks within each community, followed by community feedback sessions. Children will receive art materials packs, and artists will be appropriately remunerated for their time, expertise and cultural knowledge.

#### Cultural authority and protocols

Throughout the artwork development process, we will ensure appropriate cultural protocols are followed. This includes seeking guidance from elders and cultural knowledge holders within each community, and ensuring artists have the cultural authority to create and share visual representations. We will respect intellectual property rights and cultural ownership of designs, and follow appropriate processes for depicting cultural symbols, stories and knowledge. Necessary permissions for use of cultural imagery and designs will be obtained in accordance with community protocols and cultural governance processes.

#### Collaborative analysis and refinement

Each community and the CWG will have the opportunity to review all artwork concepts to ensure cultural appropriateness, authenticity and alignment with the well-being items. Children from each community will be engaged in final review sessions to provide feedback on which visual representations best capture the meaning of each item within their cultural context.

#### Collaborative analysis

At critical points during the artwork co-design and feedback process, the CWG will be convened to inform the iterative development of the unified artwork version and to ensure the final measure appropriately celebrates and represents Aboriginal and Torres Strait Islander peoples from diverse cultural groups across Australia.

### Phase 4: Develop and validate the pictorially-supported descriptive system

In Phase 4, guided by the CWG, we will build on best practice measure development approaches to incorporate validation of items with accompanying pictures to test the draft illustrated well-being items (developed in Phases 2 and 3) and develop the descriptive system for the measure. This phase will use quantitative methods to validate the measure through children experiencing it in practice, with the artwork accompanying all items.

#### Knowledge holders, recruitment and data collection

The items developed in Phases 2 and 3 will be included in an online survey for Aboriginal and Torres Strait Islander children living in metropolitan, rural and remote areas. A sample of around 300 children will be recruited via partner organisations and through investigator and research team networks. A purposive sampling approach will be used to ensure diversity across age, sex and remoteness. The online survey will take around 30 min to complete. Children will enter a prize draw to win an art materials pack; a general approach that has been used successfully in our existing project with youth (NHMRC GNT1125434). This sample size will be sufficient to conduct psychometric analyses, based on our experience and our existing project with youth^23^ and adults,^24^ in which a similar sample size completed a survey with 30 candidate well-being items.

#### Statistical analysis

We will conduct classical psychometric analysis at the item and dimension level to assess reliability and validity. We will also explore differences across sociodemographic groups. Factor analysis will be undertaken to ensure the final measure includes a broad set of dimensions of well-being relevant to Aboriginal and Torres Strait Islander children. Exploratory Factor Analysis will initially be used to examine dimensionality of groups of well-being items, without imposing a pre-specified structure of dimensions. Items that do not load on any factor will be considered for exclusion from the measure alongside other criteria to ensure that crucial items (eg, identified as important in Phase 2) are not excluded. Confirmatory Factor Analysis will also be used to test the preferred dimension structure. A range of statistics will be used to assess model fit and guide dimension development. We will also use Item Response Theory methods to understand measurement characteristics of the items (including measurement discrimination and differential item functioning) and inform decision making about which items are included in the final measure.^12^

#### Exploratory testing

We will conduct exploratory measurement invariance (MI) testing to explore whether the measure functions equivalently across key sociodemographic groups (eg, age, sex, remoteness). This analysis will test whether the measure has the same meaning and structure across these groups, ensuring fair and valid assessment for all Aboriginal and Torres Strait Islander children.

MI will be evaluated hierarchically across two levels. Configural invariance tests whether the same factor structure holds across sociodemographic groups. Scalar invariance tests whether item intercepts are equivalent across groups (ie, children with the same level of well-being respond similarly regardless of sociodemographic characteristics).

Model fit will be assessed using standard fit indices (Comparative Fit Index (CFI), Tucker-Lewis Index, Root Mean Square Error of Approximation (RMSEA), and Standardised Root Mean Square Residual), with changes in CFI ≤0.010 and RMSEA ≤0.015 between nested models indicating invariance.^52 53^ If full scalar invariance is not achieved, partial invariance testing will be conducted to identify any items that function differently across groups.

Additionally, we will examine differential item functioning (DIF) using Item Response Theory methods to identify whether specific items perform differently across sociodemographic groups after controlling for overall well-being levels. Items showing substantial DIF (McFadden’s pseudo R² ≥0.02) will be reviewed with the CWG to determine whether differences reflect meaningful variation or measurement bias requiring item modification.^54^

#### Collaborative analysis

We will engage the CWG using Collaborative Yarning to seek guidance before finalisation of the items, particularly when interpreting MI results and determining whether any identified differences across sociodemographic groups represent meaningful variation or measurement concerns. At the end of Phase 4, we will have a final pictorially-supported well-being measure with culturally-responsive artwork developed through community co-design and feedback, which will be taken forward to Phase 5 for preference elicitation testing.

### Phase 5: Preference scoring system development

In Phase 5, guided by the CWG, we will use Best Worst Scaling (BWS) methods to develop a preference scoring system for the well-being measure, which will be a scoring option for the measure alongside total and factor summative scoring. We will use best practice methods^55^ and follow approaches taken in our existing project with youth^23^ and other studies with children.^27^,^29^ This approach will allow development of a scoring system for each item response level to estimate overall and dimension specific preference-based well-being scores on a 0–100 scale.

#### Knowledge holder recruitment

We will recruit in the order of 400 children via our partner organisations and through investigator and team networks. They will be invited to participate in this survey through email invitations to parents/carers and direct approaches through partner organisations. A broad range of children will be included (ensuring diversity in age, sex and remoteness) to enable us to examine any differences across sociodemographic and geographical groups. The BWS survey will take approximately 20 min to complete. Partner organisations include community-controlled health services, schools, and Aboriginal and Torres Strait Islander community organisations engaged through our existing networks. Survey items will be randomised in presentation order to minimise order effects.

#### Data collection

The BWS survey will be administered online, and children will enter a draw to win a prize. The survey will consist of around 10–12 questions which present two well-being item/response category statements, based on the final measure developed in Phase 4. Children will be asked to choose which statement they think is the ‘best’ in relation to how it would make them feel, that is, their well-being. We have successfully used this method previously with adults and youth; Ratcliffe *et al* have used this approach with slightly older children in CHU9D (Child Health Utility 9D) development.^27^,^29^

#### Sample size

The optimal sample size for a BWS survey is dependent on the final number of well-being items and response levels to be included in the descriptive system.^56^ Based on our previous measure development, we anticipate that there will be 16–18 items in the final measure. It is not feasible to present all combinations of well-being items to all children, therefore we will use a balanced incomplete block design.^57^ Given large sample properties can be achieved with around 50 respondents per block or version,^27 29 55 58^ a design with 8 blocks and around 50 respondents/block (n~400) will be robust enough to estimate main effects, first order interactions and examine differences between subgroups. This sample size is also consistent with previous successful applications of this method.^2729 58^,^61^

#### Statistical analysis

The relative importance of all items and response levels (referred to as statements) is given by the regression coefficients from a multinomial logit regression model. The absence or presence of a statement in a BWS task is coded as 0 or 1. For regression analysis the statement chosen as ‘best’ in each question will be coded as ‘1’ while the item chosen as the ‘worst’ is coded as ‘−1’. Since these values are on the same interval scale, a linear transformation can be applied to the dimension level values to ensure that the highest well-being state (ie, the sum of the best level of all dimensions) has a value of 100 and the lowest well-being state has a value of 0.

#### Collaborative analysis

At critical points during the statistical analysis process, the CWG will be convened to inform the iterative development of the final scoring.

## Ethics and dissemination

Ethics approvals have been obtained for this study from: the University of Queensland Human Research Ethics Committee (Ref: 2023/HE000607); Western Australian Aboriginal Health Ethics Committee (Ref: WAHREC1260); Far North Queensland Human Research Ethics Committee (Ref: 2023/QCH/99346); Aboriginal Health and Medical Research Council of New South Wales (Ref: 2140/23); Aboriginal Health Council of South Australia (Ref: 04–23-1063); Menzies School of Health Research and Northern Territory Department of Health Human Research Ethics Committee (Ref: HREC 2023–4608); and the Australian National University Human Research Ethics Committee (Ref: H/2024/0914). Approvals from the Departments of Education were obtained in each state and territory where school sites were engaged in the project. Each team member attending sites attained the relevant jurisdictions’ Working with Children Check. Results will be published in international peer-reviewed journals and presented at conferences, and summaries will be provided to the study partner organisations and other relevant organisations for broader dissemination. Children will receive study communications via their partner organisations in appropriate formats.

## Methodological limitations

Methodological limitations of this study include: the potential difficulty of recruiting sufficiently large and geographically diverse samples within proposed timelines, which will be managed through contingency recruitment plans and extended partner networks; the unproven feasibility of a 30-minute online survey for the youngest participants (aged 5–7 years), which will be addressed through piloting and adaptive design; and the challenge of applying Western psychometric validation frameworks within an Indigenist research design, which will be managed through CWG review of all interpretations to ensure community perspectives inform analytic decisions. The use of think-aloud yarns with Aboriginal and Torres Strait Islander children is novel and involves methodological adaptation, the outcomes of which will be documented and reported to support future replication.

## Data availability

The data generated during this study will be owned by Aboriginal and Torres Strait Islander communities and managed in accordance with Indigenous Data Sovereignty principles. Aggregate deidentified data from psychometric phases may be made available on reasonable request to the corresponding author, subject to community consent and data governance approval. Qualitative data from Art and Yarning and think-aloud sessions will not be publicly shared, in line with community agreements and ethical obligations.

Our team has strong experience in translating measure development into health service practice. KMA, KH, MD, AG and DG are currently conducting a project to implement the WM2A measure in health services in New South Wales (MRFF GNT2007834). The representation of senior researchers (KH and DG) on clinical and policy relevant state and national committees will ensure the uptake of the WM2K measure into research, policy and practice. Dickson is co-leading a health and well-being researcher-capacity building component of an international collaboration, Network Environments for Indigenous Health Research Canada Holistic Aboriginal and Torres Strait Islander Mental Health and Wellness. DG is also Chair of the Australian Institute of Health and Welfare Aboriginal and Torres Strait Islander Social, Emotional, Well-being Consortium. Via these links, our work will form the fundamental grounding for measuring and valuing well-being for Aboriginal and Torres Strait Islander children, a critical first step in developing a measure that can ultimately be applied and compared across national and international contexts.

## References

[1] Clarkson C (2017). Human occupation of Northern Australia by 65,000 years ago. Nature New Biol.

[2] Anderson K, Elder-Robinson E, Gall A (2022). Aspects of Wellbeing for Indigenous Youth in CANZUS Countries: A Systematic Review. Int J Environ Res Public Health.

[3] United Nations (1989). Convention on the Rights of the Child.

[4] United Nations general assembly (2007). United Nations Declaration on the Rights of Indigenous Peoples.

[5] AIHW (2018). Aboriginal and Torres Strait Islander adolescent and youth health and wellbeing 2018: in brief.

[6] Commonwealth of Australia (2020). Closing the Gap Report 2020.

[7] Azzopardi PS, Sawyer SM, Carlin JB (2018). Health and wellbeing of Indigenous adolescents in Australia: a systematic synthesis of population data. The Lancet.

[8] Commonwealth of Australia (2017). National strategic framework for aboriginal and torres strait islander peoples, mental health and social and emotional wellbeing.

[9] Dodge R, Daly A, Huyton J (2012). The challenge of defining wellbeing. Intnl J Wellbeing.

[10] Garvey G, Anderson K, Gall A (2021). The Fabric of Aboriginal and Torres Strait Islander Wellbeing: A Conceptual Model. IJERPH.

[11] Anderson K, Garvey D, Howard K (2025). Understanding wellbeing from the perspectives of First Nations Australian youth: Findings from a national qualitative study. *SSM - Mental Health*.

[12] Howard K, Garvey G, Anderson K (2024). Development of the What Matters 2 Adults (WM2A) wellbeing measure for Aboriginal and Torres Strait Islander adults. Soc Sci Med.

[13] McLean K (2022). Health, development and learning screening and assessment tools for children and young people aged 5–18 years: an evidence check rapid review brokered by the sax institute (www.saxinstitute.org.au).

[14] Jones N, Presler-Marshall E, Samuels F (2018). Empowering Adolescent Girls in Developing Countries.

[15] Toumbourou JW (2014). Review of key risk and protective factors for child development and wellbeing (antenatal to age 25).

[16] OECD (2021). Measuring what matters for child well-being and policies. https://www.oecd.org/en/publications/measuring-what-matters-for-child-well-being-and-policies_e82fded1-en.html.

[17] Varni JW, Limbers CA, Burwinkle TM (2007). How young can children reliably and validly self-report their health-related quality of life?: an analysis of 8,591 children across age subgroups with the PedsQL 4.0 Generic Core Scales. Health Qual Life Outcomes.

[18] Solans M, Pane S, Estrada M-D (2008). Health-related quality of life measurement in children and adolescents: a systematic review of generic and disease-specific instruments. Value Health.

[19] Houts PS, Doak CC, Doak LG (2006). The role of pictures in improving health communication: a review of research on attention, comprehension, recall, and adherence. Patient Educ Couns.

[20] Schubbe D, Scalia P, Yen RW (2020). Using pictures to convey health information: A systematic review and meta-analysis of the effects on patient and consumer health behaviors and outcomes. Patient Educ Couns.

[21] Green MJ, Myers KR (2010). Graphic medicine: use of comics in medical education and patient care. BMJ.

[22] Garvey G, Anderson K, Gall A (2021). What Matters 2 Adults (WM2Adults): Understanding the Foundations of Aboriginal and Torres Strait Islander Wellbeing. IJERPH.

[23] Garvey G (2024). What Matters to Aboriginal and Torres Strait Islander Youth (WM2Y): a study protocol to develop a national youth well-being measure. BMJ Open.

[24] Howard K, Anderson K, Cunningham J (2020). What Matters 2 Adults: a study protocol to develop a new preference-based wellbeing measure with Aboriginal and Torres Strait Islander adults (WM2Adults). BMC Public Health.

[25] Anderson K, Gall A, Butler T (2023). Development of Key Principles and Best Practices for Co-Design in Health with First Nations Australians. IJERPH.

[26] Boateng GO, Neilands TB, Frongillo EA (2018). Best Practices for Developing and Validating Scales for Health, Social, and Behavioral Research: A Primer. Front Public Health.

[27] Ratcliffe J, Huynh E, Chen G (2016). Valuing the Child Health Utility 9D: Using profile case best worst scaling methods to develop a new adolescent specific scoring algorithm. Soc Sci Med.

[28] Ratcliffe J, Couzner L, Flynn T (2011). Valuing Child Health Utility 9D health states with a young adolescent sample: a feasibility study to compare best-worst scaling discrete-choice experiment, standard gamble and time trade-off methods. Appl Health Econ Health Policy.

[29] Ratcliffe J, Chen G, Stevens K (2015). Valuing Child Health Utility 9D Health States with Young Adults: Insights from a Time Trade Off Study. Appl Health Econ Health Policy.

[30] National health and Medical Research Council (2018). Ethical conduct in research with Aboriginal and Torres Strait Islander Peoples and communities: Guidelines for researchers and stakeholders.

[31] Australian Institute of Aboriginal and Torres Strait Islander Studies (2012). Guidelines for Ethical Research in Australian Indigenous Studies.

[32] Alvesson M, Skoldburg K (2000). Reflexive Methodology.

[33] Nilson C (2017). A Journey Toward Cultural Competence: The Role of Researcher Reflexivity in Indigenous Research. J Transcult Nurs.

[34] Huria T (2019). Consolidated criteria for strengthening reporting of health research involving indigenous peoples: the CONSIDER statement. BMC Med Res Methodol.

[35] Rigney L-I (1999). Internationalization of an Indigenous Anticolonial Cultural Critique of Research Methodologies: A Guide to Indigenist Research Methodology and Its Principles. *Wicazo Sa Review*.

[36] Smith LT (1999). Decolonizing Methodologies: Research and Indigenous Peoples.

[37] Butler T, Gall A, Garvey G (2022). A Comprehensive Review of Optimal Approaches to Co-Design in Health with First Nations Australians. Int J Environ Res Public Health.

[38] Angell C, Alexander J, Hunt JA (2015). ‘Draw, write and tell’: A literature review and methodological development on the ‘draw and write’ research method. J Early Child Res.

[39] Noonan RJ, Boddy LM, Fairclough SJ (2016). Write, draw, show, and tell: a child-centred dual methodology to explore perceptions of out-of-school physical activity. BMC Public Health.

[40] Bessarab D, Ng’andu B (2010). Yarning About Yarning as a Legitimate Method in Indigenous Research. Int J Coop Inf Syst.

[41] Castleberry A, NVivo 10 (2014). American journal of pharmaceutical education.

[42] Shay M (2019). Extending the yarning yarn: Collaborative Yarning Methodology for ethical Indigenist education research. *AJIE*.

[43] Charters E (2003). The Use of Think-aloud Methods in Qualitative Research An Introduction to Think-aloud Methods. *Brock Education Journal*.

[44] Gall A, Howard K, Anderson K (2023). The Suitability and Acceptability of the Think-Aloud Method to Aboriginal and Torres Strait Islander Adults. Int J Qual Methods.

[45] Horwood J, Pollard B, Ayis S (2010). Listening to patients: using verbal data in the validation of the Aberdeen Measures of Impairment, Activity Limitation and Participation Restriction (Ab-IAP). BMC Musculoskelet Disord.

[46] Willis G (2006). Cognitive interviewing as a tool for improving the informed consent process. J Empir Res Hum Res Ethics.

[47] Ryan M, Watson V, Entwistle V (2009). Rationalising the ‘irrational’: a think aloud study of discrete choice experiment responses. Health Econ.

[48] Baauw E, Markopoulous P (2004). A comparison of think-aloud and post-task interview for usability testing with children.

[49] Ogden J, Roy-Stanley C (2020). How do children make food choices? Using a think-aloud method to explore the role of internal and external factors on eating behaviour. Appetite.

[50] Rozendaal E, Buijzen M, Valkenburg PM (2012). Think-Aloud Process Superior to Thought-Listing in Increasing Children’s Critical Processing of Advertising. Hum Commun Res.

[51] Someren M, Barnard Y, Sandberg J (1994). The think aloud method - a practical guide to modelling cognitiveprocesses.

[52] Cheung GW, Rensvold RB (2002). Evaluating Goodness-of-Fit Indexes for Testing Measurement Invariance. Structural Equation Modeling: A Multidisciplinary Journal.

[53] Chen FF (2007). Sensitivity of Goodness of Fit Indexes to Lack of Measurement Invariance. Struct Equ Modeling.

[54] Choi SW, Gibbons LE, Crane PK (2011). lordif : An *R* Package for Detecting Differential Item Functioning Using Iterative Hybrid Ordinal Logistic Regression/Item Response Theory and Monte Carlo Simulations. J Stat Soft.

[55] Lancsar E (2013). Best worst discrete choice experiments in health: methods and an application. Soc Sci Med.

[56] Hensher DA, Rose JM, Greene WH (2015). Applied Choice Analysis.

[57] Rose John M, Michiel CJB, David AH, Kenneth JB (2007). Handbook of Transport Modelling.

[58] Ratcliffe J, Flynn T, Terlich F (2012). Developing adolescent-specific health state values for economic evaluation: an application of profile case best-worst scaling to the Child Health Utility 9D. Pharmacoeconomics.

[59] Viney R, Norman R, Brazier J (2014). An Australian discrete choice experiment to value eq-5d health states. Health Econ.

[60] Huynh E (2025). Australian Scoring Algorithms for the Pediatric Quality of Life Inventory™ Generic Core Scales: Best-Worst Scaling and Discrete Choice Experiment.

[61] Logeman C, Guha C, Howell M (2020). Developing Consensus-Based Outcome Domains for Trials in Children and Adolescents With CKD: An International Delphi Survey. Am J Kidney Dis.

