# Supplementary material for: What Matters 2 Kids: a mixed-methods protocol for developing a culturally-responsive illustrated well-being measure for Aboriginal and Torres Strait Islander children
Source: BMJ Open. 2026 Jun 18;16(6):e115255. doi: 10.1136/bmjopen-2025-115255 (PMC13288972; doi:10.1136/bmjopen-2025-115255)
Supplement: Supplementary file 1 [file bmjopen-2025-115255.reviewer_comments.pdf]

PEER REVIEW HISTORY

BMJ Open publishes all reviews undertaken for accepted manuscripts. Reviewers are asked to complete a checklist review form and are provided with free text boxes to elaborate on their assessment. These free text comments are reproduced below.

ARTICLE DETAILS

Title (Provisional)

What Matters 2 Kids: A Mixed-Methods Protocol for Developing a Culturally-Responsive Illustrated Wellbeing Measure for Aboriginal and Torres Strait Islander Children

Authors

Anderson, Kate Mallory; Howard, Kirsten; Dickson, Michelle; Gall, Alana; Garvey, Darren; Howell, Martin; Theobald, Maryanne; Mulhern, Brendan James; Viney, Rosalie; Garvey, Gail

---

VERSION 1 - REVIEW

**Reviewer**	**1**
**Name**	**Barwick, Melanie**
**Affiliation**	**Hospital for Sick Children, Research Institute**
**Date**	**17-Apr-2026**
**COI**	**None**

This is a strong, thoughtful, and ambitious study protocol addressing an important gap in the measurement of wellbeing for Aboriginal and Torres Strait Islander children. The protocol demonstrates considerable strengths in Indigenous leadership, ethical integrity, cultural grounding, and methodological sophistication. However, several areas would benefit from greater methodological specificity, justification of feasibility, and conceptual clarity—particularly where aspirational principles are not fully operationalized.

Strengths of the study

- Strong Aboriginal and Torres Strait Islander leadership, governance, and authorship, including a majority-Indigenous Co-Design Working Group guiding all phases of the research.
- Explicit alignment with Indigenist methodologies, strengths-based approaches, and the CONSIDER statement, reinforcing ethical rigour and cultural integrity.
- Clear and compelling rationale for a culturally grounded child wellbeing measure that reflects Indigenous relational worldviews.
- Innovative, child-centred methods (Art and Yarning, think-aloud yarns) that are culturally and developmentally appropriate for children aged 5–11 years.
- Novel integration of culturally responsive, co-designed artwork as an integral component of the wellbeing measure, rather than as a supplementary aid.

- Methodologically rigorous mixed-methods design, linking qualitative concept elicitation, psychometric validation, and preference-based scoring.
- The inclusion of preference-based scoring enhances downstream utility for policy, planning, and resource allocation.
- Comprehensive ethics approvals and strong dissemination pathways, including free public access to the final measure, although unclear where it will be housed or how it will be disseminated.

Limitations and areas requiring clarification
Conceptual and methodological clarity

- The protocol describes the study as *pioneering* while also noting that methods build on prior *What Matters* measure development studies.
    - It is unclear what is substantively new and what is adapted from earlier work.
    - Clarification is needed on whether this is the first *What Matters* study to formally co-design and embed artwork as a core measurement component.
- Indigenist methodologies are described largely in aspirational terms.
    - The protocol would be strengthened by clearer articulation of how these methodologies are operationalized in practice, not just what they seek to accomplish.
    - Readers would benefit from concrete examples of how Indigenist principles shape governance, data interpretation, and analytic decision-making—particularly where Western psychometric methods are employed.

Governance, power-sharing, and process transparency

- Recruitment processes are insufficiently specified across study phases.
    - Greater detail is needed regarding how children, community researchers, artists, and Co-Design Working Group (CWG) members will be identified and recruited, and by whom.
- The protocol would benefit from an explicit description of governance structures and standard operating procedures (SOPs), including:
    - Conflict resolution mechanisms;
    - Processes to ensure equitable power distribution;
    - How disagreements are managed within the CWG or between communities and academics.
    - Whether co-development principles will be assessed? [good opportunity to do so]
- Further clarification is needed regarding:
    - How decisions are made when consensus cannot be reached;
    - How community priorities may override pre-specified research plans.
- More detail is needed on how the CWG itself is established and maintained:
    - Nomination and appointment processes;
    - Roles and tenure across phases;
    - Onboarding, orientation, and accountability to communities.

Curious about the health economics positioning

- The protocol includes several health economists, yet the study's economic contribution is not clearly articulated early on.
    - The study appears to be primarily a measure development protocol rather than an economic evaluation.
    - Clarification is needed that the role of health economics is to enable **future economic evaluation and policy-relevant valuation**, not to conduct cost-effectiveness analysis within this study.

- Making this explicit would help align reader expectations with the multidisciplinary team composition.

Recruitment feasibility and timelines

- The proposed recruitment numbers (approximately 150 children in Phase 2, 300 in Phase 4, and 400 in Phase 5) raise questions about feasibility.
  - The protocol would benefit from clearer justification of these targets, drawing on prior recruitment experience.
  - A recruitment timeline for each phase, including whether phases overlap, should be specified.
  - Contingency plans for if recruitment targets are not met would strengthen confidence in the feasibility.

Feasibility for young children

- The protocol proposes a 30-minute online survey for children aged 5–11 years.
  - The feasibility of this duration—particularly for younger children (5–7 years)—is unclear.
  - Evidence or prior experience supporting this assumption should be provided.
  - Strategies to manage fatigue, disengagement, or partial completion should be described, along with data quality thresholds.

Analytical methods clarification

- The term **"error coding"** in think-aloud yarn analysis is not defined.
  - Clarification is needed regarding what constitutes an "error" (e.g., comprehension, recall, judgment, response mapping).
  - It should be explained how error coding is applied in a developmentally appropriate, non-deficit-based manner.
  - The relationship between error coding and thematic analysis should be explicitly described.

Equity, sex, and gender considerations

- The protocol indicates analyses will consider age, sex, and remoteness, but does not address gender.
  - Clarification is needed regarding whether gender identity (distinct from sex assigned at birth) will be collected or considered.
  - If gender data are not included, justification should be provided, particularly given the study's equity commitments.

Language and decolonizing considerations

- The repeated use of the term *"stakeholder"* is potentially misaligned with Indigenist and decolonizing research principles.
  - From a Canadian Indigenous research perspective, this term is increasingly replaced with *knowledge holder*, *knowledge user*, *interest holder*, or *rights holder*.
  - The authors may wish to revise terminology or acknowledge jurisdictional preferences in language use.

Clarity of the final product

- While the development process is well described, the protocol provides a limited sense of the end-user product.
  - Including a mock-up, conceptual rendering, or screenshot from an existing What Matters measure (clearly labelled as illustrative) would help readers understand:
    - How artwork and text are integrated;
    - The format (digital/paper);
    - How children will interact with the measure in practice.

Summary

This is a high-quality, ethically grounded, and potentially highly impactful study protocol. Addressing the points above—primarily through clarification rather than substantive redesign—would substantially strengthen the manuscript, improve transparency, and enhance its methodological credibility for an international readership.

VERSION 1 - AUTHOR RESPONSE

PART B: REVIEWER COMMENTS (Reviewer 1 — Dr. Melanie Barwick)

We thank Dr. Barwick for her detailed and constructive review. Her comments have substantially improved the methodological clarity of the manuscript.

Reviewer Comment 1: Conceptual novelty

Comment: Clarify what is substantively new in this study versus what has been adapted from prior What Matters studies, particularly regarding the artwork component.

> **Response:** The Indigenist Methodology section now explicitly states that this is the first What Matters study to formally co-design and embed culturally-responsive artwork as a core measurement component (rather than as a supplementary aid). This distinction is also reflected in the Strengths and Limitations section. See pp. 7 and 3.

Reviewer Comment 2: Indigenist methodologies — operationalisation

Comment: Indigenist methodologies are described in aspirational terms. Provide concrete examples of how these principles are operationalised in governance, data interpretation, and analytic decision making.

> **Response:** The Indigenist Methodology section has been expanded with concrete examples of how Indigenist principles operate in practice, including: privileging Aboriginal and Torres Strait Islander voices in analytic interpretation via Collaborative Yarning; ensuring recruitment and data collection is led or co-led by Aboriginal and Torres Strait Islander researchers; and embedding community review at key decision points in item development, artwork refinement, and psychometric interpretation. See p. 7.

Reviewer Comment 3: Governance, power sharing, and process transparency

Comment: Provide greater detail on CWG recruitment and nomination, governance structures and SOPs, conflict resolution, equitable power distribution, and what happens when consensus cannot be reached.

> **Response:** Substantial governance detail has been added to the Indigenist Methodology section, covering: CWG member identification through partner consultation; agreed roles and onboarding at project commencement; governance principles including equitable power distribution and transparent decision making; an escalation pathway when consensus cannot be reached (with community priorities taking precedence); and documentation of conflict resolution processes in the project governance framework. See p. 7.

Reviewer Comment 4: Health economics positioning

Comment: Clarify that health economists' role is to enable future economic evaluation, not to conduct cost-effectiveness analysis within this study.

> **Response:** A clarifying statement has been added to the Phase 5 section confirming that the role of health economics is to enable preference-based scoring for future economic evaluation; cost-effectiveness analysis is not within the scope of the current study. See p. 15.

Reviewer Comment 5: Recruitment feasibility and timelines

Comment: Justify recruitment targets, provide a timeline, and describe contingency plans.

> **Response:** Recruitment targets are now grounded in prior experience from the WM2Adults and WM2Youth studies. Contingency plans (extended recruitment period, additional partner activation, investigator network outreach) and a minimum data completeness threshold (80%) have been added to Phase 4. Overall project dates serve as the timeline reference. See pp. 3 and 13–14.

Reviewer Comment 6: Feasibility of 30-minute survey for young children

Comment: Address feasibility of the survey duration for children aged 5–7 years, and describe strategies to manage fatigue and partial completion.

> **Response:** Phase 4 now states that feasibility for younger children will be monitored closely. Strategies added include adaptive survey pathways, item randomisation to reduce order effects, multi-session completion options, and a data completeness threshold. See p. 3 (S&L bullet 5) and pp. 13–14.

Reviewer Comment 7: Error coding — definition and application

Comment: Define "error coding", clarify what constitutes an error, explain how it is applied in a strengths-based way, and describe its relationship to thematic analysis.

> **Response:** A definition of error coding has been added to the Phase 2b analysis section, specifying the three error types (recall, judgement, response mapping), clarifying its strengths-based application (focusing on item-level issues, not child deficits), and describing how it feeds into the subsequent thematic analysis step. See p. 11.

Reviewer Comment 8: Equity, sex, and gender considerations

Comment: Address gender identity (distinct from sex), and provide justification if not collected.

> **Response:** Phase 4 now confirms that gender identity (as distinct from sex) will be collected via self-report alongside age, sex, and remoteness, with a note acknowledging the importance of gender-affirming approaches. Exploratory analyses by gender identity will be conducted where cell sizes allow. See p. 14.

Reviewer Comment 9: Terminology — "stakeholder"

Comment: Replace "stakeholder" with more appropriate terminology aligned with Indigenist and decolonising research principles.

> **Response:** "Stakeholders" has been replaced with "knowledge holders" throughout the manuscript, in both the Indigenist Methodology and Community Involvement sections. See pp. 7–8.

Reviewer Comment 10: Clarity of the final product

Comment: Include a mock-up or conceptual rendering to help readers understand how artwork and text are integrated in the final measure.

> **Response:** As artwork co-design occurs in Phase 3 (not yet commenced), a finalised or illustrative mock-up is not available at this protocol stage. This has been acknowledged in the Strengths and Limitations section. The existing What Matters measures (cited as references 23 and 24) may assist readers familiar with the broader program. Visual examples will be included in publications reporting study findings.

VERSION 2 - REVIEW

**Reviewer**	**1**
**Name**	**Barwick, Melanie**
**Affiliation**	**Hospital for Sick Children, Research Institute**
**Date**	**11-May-2026**
**COI**	

This is incredibly innovative work, and I look forward to the outcomes. I'm appreciative that the initial concerns have all been addressed. There are several remaining grammatical issues. I recommend a final review of language and grammar, potentially using standard editing tools (e.g., Grammarly) or professional editing services, to improve readability and consistency. Also, the Methodological Limitations should begin in its own paragraph. All the best in conducting this research.

VERSION 2 - AUTHOR RESPONSE

PART B: REVIEWER COMMENTS (Reviewer 1 - Dr. Melanie Barwick)
We thank Dr. Barwick for her continued engagement with this manuscript and her constructive feedback. Her comments have further improved the clarity and quality of the manuscript.

Reviewer Comment 1: Grammatical issues
Comment: There are several remaining grammatical issues. The reviewer recommended a final review of language and grammar, potentially using standard editing tools (e.g., Grammarly) or professional editing services, to improve readability and consistency.
Response: The manuscript has been reviewed carefully and eleven grammatical corrections have been made throughout. These include: (1) insertion of a missing comma after 'colonisation' before the relative clause 'which continue to cause' (Background); (2) insertion of a missing full stop after 'previous studies' before 'Inclusion criteria' (Phase 2a); (3) correction of an incomplete parallel construction in the think-aloud yarns elicitation list from '3) any topics or item phrasing that are appropriate' to '3) there are topics or item phrasing that are appropriate' (Phase 2b); (4) correction of 'consisting of a majority Aboriginal and Torres Strait Islander knowledge holders' to 'consisting of a majority of Aboriginal and Torres Strait Islander knowledge holders' (Indigenist Methodology); (5) correction of 'centering' to 'centring' (Australian English; Indigenist Methodology); (6) correction of 'is comprised of' to 'comprises' (Phase 2a); (7) removal of the redundant preposition 'in' from 'experience in working with children' (Phase 3); (8–9) correction of two sentence fragments - 'Configural invariance to test whether…' and 'Scalar invariance to test whether…' - to full sentences with finite verbs (Phase 4); (10) correction of the run-on sentence 'We have successfully used this method previously with adults, youth and Ratcliffe et al have used this approach' to 'We have successfully used this method previously with adults and youth; Ratcliffe et al. have used this approach' (Phase 5); (11) removal of a double space and correction of 'has a value 0' to 'has a value of 0' (Phase 5). All corrections are shown as tracked changes throughout the manuscript.

Reviewer Comment 2: Methodological Limitations paragraph
Comment: The Methodological Limitations should begin in its own paragraph.
Response: The Methodological Limitations text has been separated from the preceding Ethics and Dissemination paragraph and now begins its own clearly delineated paragraph under a dedicated 'Methodological Limitations' section heading, consistent with the formatting of other subsections. See p. 17.
